# The endogenous opioid system in the medial prefrontal cortex mediates ketamine’s antidepressant-like actions

**DOI:** 10.1038/s41398-024-02796-0

**Published:** 2024-02-12

**Authors:** Cheng Jiang, Ralph J. DiLeone, Christopher Pittenger, Ronald S. Duman

**Affiliations:** 1 Department of Psychiatry, Yale University School of Medicine, New Haven, CT USA; 2 Department of Neuroscience, Yale University School of Medicine, New Haven, CT USA; 3 Department of Psychology, Yale University School of Arts and Sciences, New Haven, CT USA; 4 Child Study Center, Yale University School of Medicine, New Haven, CT USA; 5 Center for Brain and Mind Health, Yale University School of Medicine, New Haven, CT USA; 6 Department of Pharmacology, Yale University School of Medicine, New Haven, CT USA

**Keywords:** Depression, Pharmacodynamics

## Abstract

Recent studies have implicated the endogenous opioid system in the antidepressant actions of ketamine, but the underlying mechanisms remain unclear. We used a combination of pharmacological, behavioral, and molecular approaches in rats to test the contribution of the prefrontal endogenous opioid system to the antidepressant-like effects of a single dose of ketamine. Both the behavioral actions of ketamine and their molecular correlates in the medial prefrontal cortex (mPFC) are blocked by acute systemic administration of naltrexone, a competitive opioid receptor antagonist. Naltrexone delivered directly into the mPFC similarly disrupts the behavioral effects of ketamine. Ketamine treatment rapidly increases levels of β-endorphin and the expression of the μ-opioid receptor gene (*Oprm1*) in the mPFC, and the expression of gene that encodes proopiomelanocortin, the precursor of β-endorphin, in the hypothalamus, in vivo. Finally, neutralization of β-endorphin in the mPFC using a specific antibody prior to ketamine treatment abolishes both behavioral and molecular effects. Together, these findings indicate that presence of β-endorphin and activation of opioid receptors in the mPFC are required for the antidepressant-like actions of ketamine.

## Introduction

Ketamine, a noncompetitive antagonist of *N*-methyl-D-aspartate glutamate receptors (NMDARs), has been found to have rapid antidepressant and anti-suicide effects in many patients, and similar rapid antidepressant-like effects in preclinical models [1, 2]. In addition to its well-established ability to block NMDARs, ketamine also interacts with a range of other targets, including opioid receptors [3, 4]. The endogenous opioid system consists of the μ-, δ-, and κ- opioid receptors (ORs), encoded by the *Oprm1*, *Oprd1*, and *Oprk1* genes; their primary endogenous ligands are the opioid peptides β-endorphin, enkephalins, and dynorphins, respectively. These receptors and peptides are implicated in a wide range of physiological and pathological processes, including the regulation of pain and of reward and stress responses [5–7]. Accumulating evidence implicates the endogenous opioid system in the pathophysiology and treatment of depression [8]. In particular, β-endorphin is implicated in both major depressive disorder (MDD) and its treatment [9], and the μ-OR differs in multiple brain regions between healthy controls and patients with MDD [10, 11]. Previous preclinical studies extensively documented the role of medial prefrontal cortex (mPFC) in regulating ketamine’s antidepressant-like effects, implicating synaptic protein synthesis in the mPFC in ketamine’s actions [12, 13]. However, no study to date has examined the involvement of endogenous opioid signaling in the mPFC in ketamine’s behavioral and molecular effects.

In a seminal clinical study, Williams et al. reported that pretreatment with naltrexone, an opioid receptor antagonist, attenuates the antidepressant and anti-suicidal effects of ketamine in depressed patients [14, 15] (although another clinical study did not show such an effect in patients treated with naltrexone for alcohol use [16]). In preclinical work, systemic naltrexone pretreatment was found to block the antidepressant-like effects of ketamine in three studies [17–19], but not in a fourth [20]. Here, we test the hypothesis that β-endorphin and activation of opioid receptors in the mPFC is required for the antidepressant-like actions of ketamine, and for the underlying molecular mechanisms in the mPFC.

## Materials and methods

### Animals

Adult male Sprague-Dawley rats (Charles River Laboratories; 250–500 g at the time of experiments) were obtained and allowed at least one week acclimation to housing facilities. Rats were singly housed at least 5 days before behavioral experiments or tissue collection and maintained on a 12 h light/dark cycle, with *ad libitum* access to food and water. Rats were randomly assigned to different experimental groups with age and weight matched as closely as possible. Animal use and procedures were in accordance with NIH guidelines and approved by the Yale University Animal Care and Use Committees.

### Experimental design

Experiments 1: To examine the effects of systemic opioid receptor blockade on ketamine’s behavioral actions, rats received naltrexone (Sigma-Aldrich #N3136, St. Louis, Missouri; 20 mg/kg, i.p.) or saline injection 30 min prior to ketamine (Sigma-Aldrich #K2753; 10 mg/kg, i.p.) or saline treatment. Naltrexone at this dose has been frequently used in preclinical studies [21–23], although it may lose some of its selectivity for the μ-OR and bind to δ-, and κ-ORs at this dose [24–27]. Behavioral testing was carried out starting 24 h after ketamine treatment. The same rats underwent forced swim test, female urine sniffing test, novelty suppressed feeding test, and locomotor activity test, with a 24-h interval between each test. Each group (saline/saline, saline/ketamine, naltrexone/saline, naltrexone/ketamine) consisted of 8 rats.

Experiment 2: To examine the effects of local opioid receptor blockade in the mPFC on ketamine’s behavioral actions, mPFC-cannulated rats were bilaterally infused with naltrexone (20 µg/0.5 µl/side) 30 min prior to ketamine (10 mg/kg, i.p.) or saline treatment. This naltrexone dose was chosen based on previous studies demonstrating the effects of intracranially microinjected naltrexone [28, 29]. Behavioral testing was carried out as described above. Saline/saline, saline/ketamine, and naltrexone/ketamine groups consisted of 11 rats each, while there were 10 rats in the naltrexone/saline group.

Experiment 3: ELISA and qPCR were performed with mPFC and hypothalamus collected 1 or 24 h following ketamine (10 mg/kg, i.p.) or saline treatment to examine ketamine’s effects on the presence of β-endorphin and expression of opioid receptors in the mPFC and on the expression of β-endorphin precursor, proopiomelanocortin (POMC), in the hypothalamus. Number of rats used for each analysis are listed in the Figure Legend as well as in Supplemental Table 1. One sample from each of saline and ketamine group in the 1 h mPFC *Oprm1* mRNA analysis, one sample from saline group in the 1 h mPFC *Oprk1* mRNA analysis, and one sample from ketamine group in the 24 h mPFC *Pomc* mRNA analysis were excluded due to being outliers determined by Grubb’s test.

Experiment 4: To examine the effects of local neutralization of β-endorphin on ketamine’s behavioral, mPFC-cannulated rats were bilaterally infused with anti-β-endorphin antibody (0.5 µg/0.5 µl/side; Phoenix Pharmaceuticals #G-022-33, Burlingame, California) 30 min prior to ketamine (10 mg/kg, i.p.) or saline treatment. Behavioral testing was carried out as described above. IgG/saline, anti-β-endorphin antibody/saline, and anti-β-endorphin antibody/ketamine groups consisted of 7 rats each, while IgG/ketamine group consisted of 8 rats.

Experiment 5: To examine the effects of systemic opioid receptor blockade and local neutralization of β-endorphin on ketamine’s molecular actions, rats received treatments as described in Experiments 1 and 4, and mPFC was collected 1 h or 24 h after ketamine treatment for Western blot analysis. Number of rats used for each analysis are listed in the Figure Legend and Supplementary Table 1.

### mPFC cannulation and infusion procedures

Rats were anaesthetized with a mixture of ketamine (75 mg/kg) and xylazine (5 mg/kg), and bilateral guide cannulae (P1 Technologies Inc., Roanoke, Virginia: 22-gauge) were implanted 0.5 mm above the infusion site at the following coordinates: from bregma: +3.0 mm anterior/posterior; +/−1.0 mm medial/lateral; and −4.0 mm dorsal/ventral. Following 9–14 days of recovery, rats were bilaterally infused with naltrexone or an anti-β-endorphin antibody at a rate of 0.2 µl/min, using a microinfusion pump (Harvard Apparatus, Holliston, Massachusetts). The needle was left in place for 2.5 min after the injection to allow complete dispersion of the solution. Naltrexone was dissolved in saline; control rats were infused with 0.9% saline. The anti-β-endorphin antibody was reconstituted following manufacturer’s instructions; rabbit control purified IgG (Phoenix Pharmaceuticals #NRG-500) was used as a control.

### Forced Swim Test (FST)

FST was conducted as previously described [30]. Rats were exposed to a 15-min pre-swim in 25 ± 1 °C water in a Plexiglas cylinder (65 cm height, 30 cm diameter). 24 h following the pre-swim, rats were treated as described above; 24 h following treatment they were placed in the swim tank for 10 min. Data were analyzed by scoring immobility time during minutes 2–6 in a blinded manner.

### Female Urine Sniffing Test (FUST)

FUST was conducted as previously described [30]. Rats were habituated to a sterile cotton-tipped applicator placed into their home cage for 1 h, and then exposed to a water-dipped cotton-tipped applicator for 5 min. After a 45-min interval, rats were exposed to a cotton-tipped applicator infused with 75 μl fresh urine from females of the same strain for 5 min, during which the time spent sniffing the cotton-tipped applicator was measured in a blinded manner. Time spent biting the cotton-tip was excluded from analysis.

### Novelty Suppressed Feeding Test (NSFT)

NSFT was conducted as previously described [30]. Rats were food deprived for at least 20 h and then placed in an open field with one food pellet in the center. The latency to feed was measured, with a cut-off time of 15 min. After NSFT, home cage feeding during a 15-min period was measured to verify motivation to feed.

### Locomotor Activity Test (LMA)

Rats were placed in testing cages (46 cm × 23 cm × 20 cm) for 30 min, during which the number of beam breaks was measured using Med-PC software (Med Associates, Fairfax, Vermont).

### Protein and RNA sample preparation, Western Blot, and qPCR analysis

Crude synaptosomal fraction or total homogenate of rat mPFC were prepared and analyzed by Western blot. The following primary antibodies phospho-OPRM1 (Ser375, Bioss, #BS3742R, 1:1000), total OPRM1 (Thermo Scientific, #PA124628, 1:1000), phospho-GluR1 (Ser845, Cell Signaling, #8084, 1:1000), total GluR1 (Cell Signaling, #13185, 1:1000 or DSHB, #N355/1, 1:100), and GAPDH (Cell Signaling, #2118, 1:2000 or Millipore-Sigma, #MAB374, 1:2000), as well as horseradish peroxidase–conjugated anti-rabbit or anti-mouse secondary antibody (Vector Laboratories, #PI-1000-1 or #PI-2000-1, 1:5000 to 1:10000) were used. RNA from mPFC or hypothalamus was extracted by RNeasy Mini Kit (Qiagen #74104, Hilden, Germany), reverse transcribed and subjected to qPCR, as detailed in Supplemental Material.

### Primary hypothalamic culture and in vitro ketamine treatment

Primary hypothalamic culture prepared from E18 embryos was treated with 0.5 µM ketamine on day 12 in vitro, as detailed in Supplemental Material.

### β-endorphin ELISA analysis

Rat mPFC was collected 1 h or 24 h following ketamine injection and homogenized in PBS. β-endorphin levels were measured using QuickDetect™ beta-Endorphin (Rat) ELISA Kit (BioVision, Milpitas, California #E4460) according to the manufacturer’s instructions. Protein concentrations in each sample were measured using a Pierce BCA Protein Assay Kit (Thermo Scientific, Waltham, Massachusetts) and results are presented as pg of β-endorphin in 1 mg of protein in the sample.

### Statistical analysis

Experimental sample sizes were estimated on the basis of published studies, although no statistical methods were used to predetermine sample sizes. Each experiment was replicated in at least two cohorts under our experimental conditions. All values presented were from biological replicates. Statistical analyses were performed using GraphPad Prism (San Diego, California). Values were excluded only if they were detected as outliers by Grubb’s test. This criterion was pre-established. Normal distribution and equal variances between groups were tested for each experiment. Comparisons between two groups were made using Student’s *t* test or Welch’s *t* test (when unequal variance was detected; Supplementary Fig. S3E). Correlation was calculated using Pearson’s r. Comparisons for four groups were made using two-way analysis of variance (ANOVA) followed by Sidak’s multiple comparisons, as indicated in the Figure Legends and Supplementary Material (Supplementary Table S1). All tests are two-sided. All data are presented as mean ± s.e.m.. Statistical significance is represented as asterisks at *p* values <0.05 (*), <0.01 (**), and <0.001 (***).

## Results

### Systemic naltrexone pretreatment blocks the antidepressant-like actions of ketamine

**Fig. 1:**
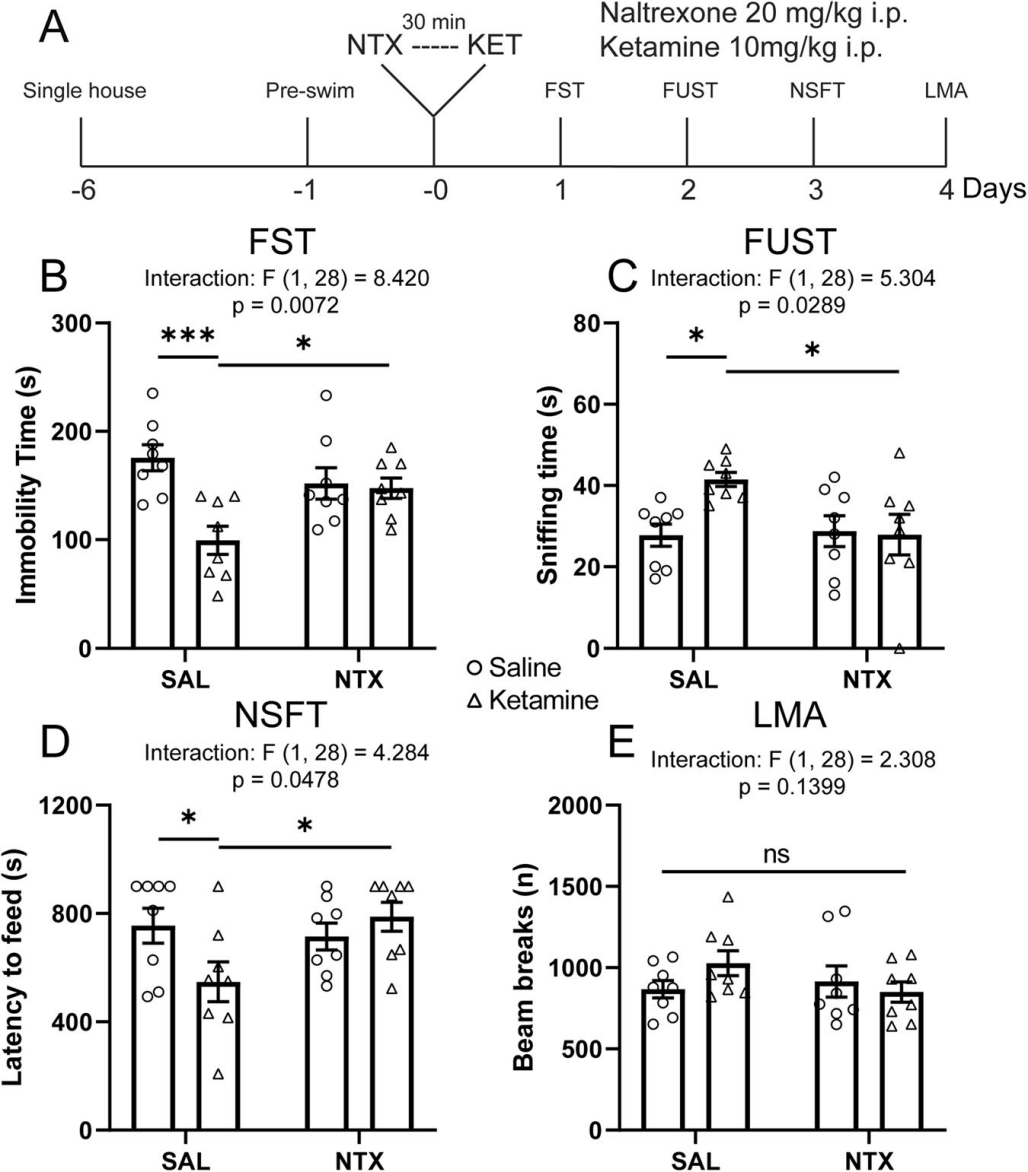
Systemic naltrexone pretreatment blocks the antidepressant-like actions of ketamine. **A** Schematic timeline of systemic naltrexone pretreatment, ketamine treatment, and behavioral testing. Systemic naltrexone pretreatment blocks (**B**) the reduction in immobility time in the FST (ketamine x naltrexone interaction *F*_(1, 28)_ = 8.420, *p* = 0.0072; see Supplementary Table S1 for full statistical results), **C** the increase in sniffing time in the FUST (interaction *F*_(1, 28)_ = 5.304, *p* = 0.0289), and (**D**) the reduction of latency to feed in the NSFT induced by ketamine (interaction *F*_(1, 28)_ = 4.284, *p* = 0.0478), without affecting locomotor activity (interaction *F*_(1, 28)_ = 2.308, *p* = 0.1399) (**E**). Two-way ANOVA followed by Sidak’s post hoc test. *n* = 8/group. Post hoc significant effects indicated as: *p < 0.05, ***p < 0.001. ns nonsignificant, SAL saline, NTX naltrexone, FST forced swim test, FUST female urine sniffing test, NSFT novelty suppressed feeding test, LMA locomotion.

To investigate whether the endogenous opioid system is required for the antidepressant-like actions of ketamine, we treated rats with naltrexone (20 mg/kg, i.p.) 30 min before ketamine (10 mg/kg, i.p.; Fig. 1A). Rats were tested in a series of behavioral paradigms, including forced swim test (FST), female urine sniffing test (FUST), novelty suppressed feeding test (NSFT), and locomotor activity (LMA), starting 24 h after ketamine treatment. In the FST, a model of behavioral despair, ketamine significantly reduced immobility time in saline-pretreated rats; this effect was completely blocked by naltrexone pretreatment (Fig. 1B). In FUST, a model in which less time spent sniffing female urine indicates anhedonia in males, ketamine significantly increased female urine sniffing time in saline- but not naltrexone-pretreated rats (Fig. 1C). In the NSFT, a paradigm in which longer latency to feed is considered an anxiety-like behavior, ketamine significantly shortened latency to feed in saline- but not naltrexone-pretreated rats (Fig. 1D), without changing home cage food consumption (Supplementary Fig. S1). Locomotor activity was not affected by either naltrexone pretreatment or ketamine treatment (Fig. 1E). Together, these results suggest that activation of opioid receptors is required for the behavioral effects of ketamine.

### Intra-mPFC naltrexone infusion blocks the antidepressant-like actions of ketamine

**Fig. 2:**
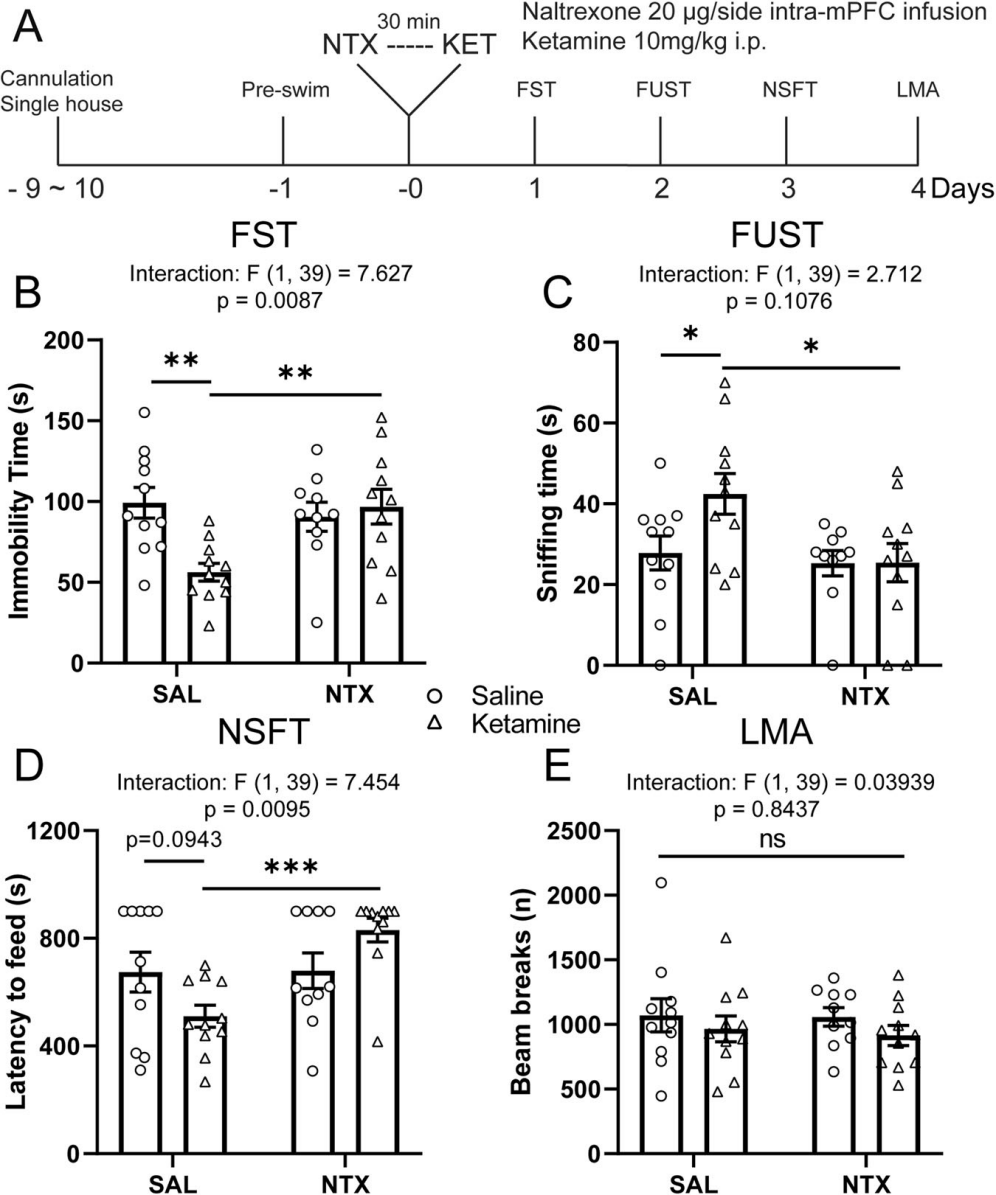
Intra-mPFC naltrexone infusion blocks the antidepressant-like actions of ketamine. **A** Schematic timeline of intra-mPFC naltrexone pretreatment, ketamine treatment and behavioral testing. Intra-mPFC naltrexone pretreatment blocks (**B**) the reduction in immobility time in the FST (ketamine x naltrexone interaction: *F*_(1, 39)_ = 7.627, *p* = 0.0087), **C** the increase in sniffing time in the FUST (naltrexone main effect: *F*_(1, 39)_ = 4.927; *p* = 0.0323), and (**D**) the reduction of latency to feed in the NSFT induced by ketamine (interaction: *F*_(1, 39)_ = 7.454, *p* = 0.0095), without affecting locomotor activity (interaction: *F*_(1, 39)_ = 0.03939, *p* = 0.8437) (**E**). Two-way ANOVA followed by Sidak’s post hoc test. *n* = 11/group for SAL/SAL, SAL/KET, and NTX/KET; *n* = 10 for NTX/SAL. Post hoc significant effects indicated as: *p < 0.05, **p < 0.01, ***p < 0.001. ns nonsignificant, SAL saline, NTX naltrexone, FST forced swim test, FUST female urine sniffing test, NSFT novelty suppressed feeding test, LMA locomotion.

As mPFC plays an important role in regulating ketamine’s antidepressant-like effects [12, 13], we sought to determine whether localized blockade of ORs using targeted naltrexone infusion into the mPFC would be sufficient to attenuate the behavioral effects of ketamine. We infused naltrexone (20 μg/0.5 μl/side) or saline into the mPFC of cannulated rats 30 min before ketamine treatment (10 mg/kg, i.p.) and subjected the rats to behavioral tests starting 24 h later (Fig. 2A). In saline-pretreated rats, ketamine induced significant behavioral effects in FST (Fig. 2B), FUST (Fig. 2C), and NSFT (Fig. 2D); all effects were absent in naltrexone-pretreated rats. There were no effects of ketamine, with or without naltrexone, on home cage feeding (Supplementary Fig. S2) or locomotor activity (Fig. 2E).

### Ketamine increases β-endorphin levels in the mPFC

While naltrexone is a non-selective OR antagonist, it has the highest affinity for μ-ORs [31, 32]. β-endorphin is the primary endogenous agonist for μ-ORs, and its presence in the cortex has been documented [33].

**Fig. 3:**
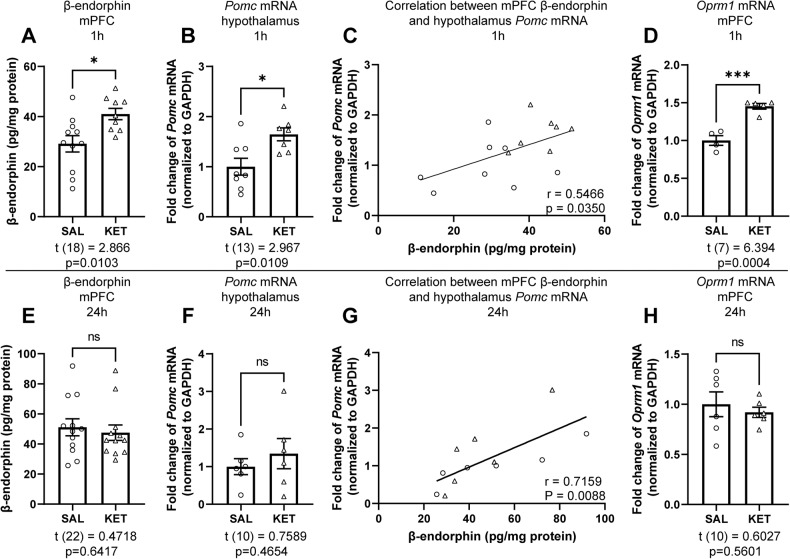
Ketamine increases endogenous opioid activity in the mPFC and hypothalamus. **A** Ketamine treatment (10 mg/kg, i.p.) significantly increases β-endorphin levels in the mPFC (*t*_(18)_ = 2.866, *p* = 0.0103; *n* = 11 for SAL, *n* = 9 for KET) and (**B**) *Pomc* mRNA expression in the hypothalamus (*t*_(13)_ = 2.967, *p* = 0.0109; *n* = 8 for SAL, *n* = 7 for KET) at 1 h. **C** Elevated β-endorphin levels in the mPFC is positively correlated with increased *Pomc* mRNA levels in the hypothalamus (*r* = 0.5466, *p* = 0.0350). **D** Ketamine treatment significantly increases *Oprm1* mRNA expression in the mPFC at 1 h (*t*_(7)_ = 6.394, *p* = 0.0004; *n* = 4 for SAL, *n* = 5 for KET). **E** At 24 h, there is no change in β-endorphin levels in the mPFC (*t*_(22)_ = 0.4718, *p* = 0.6417; *n* = 12/group) and (**F**) there is no significant increase in *Pomc* mRNA in the hypothalamus (*t*_(10)_ = 0.7589, *p* = 0.4654; *n* = 6/group); **G** but its significant correlation with mPFC β-endorphin levels remains (*r* = 0.7159, *p* = 0.0088). **H** Ketamine treatment does not change *Oprm1* mRNA expression in the mPFC at 24 h (*t*_(10)_ = 0.6027, *p* = 0.5601; *n* = 6/group). Student’s *t* test for **A**, **B**, **D**, **E**, **F**, **H**. Pearson’s r for **C**, **G**. *p < 0.05, **p < 0.01, ***p < 0.001. ns nonsignificant, SAL saline, KET ketamine.

Ketamine significantly increased β-endorphin levels in the mPFC, measured in dissected tissue using ELISA, 1 hour after treatment (Fig. 3A). β-endorphin is derived from a precursor protein, proopiomelanocortin (POMC), encoded by the *Pomc* gene, which is primarily expressed in the arcuate nucleus (ARC) of hypothalamus, as well as in the pituitary gland [34]. To investigate whether ketamine concurrently influences *Pomc* expression in the hypothalamus, we measured *Pomc* mRNA using qPCR in tissue collected 1 h following ketamine treatment. Ketamine significantly increased hypothalamic *Pomc* mRNA relative to saline-injected controls (Fig. 3B); change in *Pomc* was positively correlated with the increased β-endorphin level in the mPFC (Fig. 3C). In further support of this finding, increased β-endorphin, measured by ELISA, was released into the supernatant after ketamine treatment in hypothalamic neuronal cultures, at trend level (Supplementary Fig. S3A, B). *Pomc* expression has been previously detected in the rat cortex [35]. To determine whether this increase in mPFC β-endorphin is produced by local transcription of *Pomc*, we measured the *Pomc* mRNA levels in the mPFC 1 h after ketamine treatment and found that ketamine did not influence the *Pomc* mRNA expression in the mPFC (Supplementary Fig. S3C). Ketamine also significantly increased mPFC *Oprm1* mRNA (Fig. 3D), but not *Oprk1* mRNA (Supplementary Fig. S3D), at 1 h.

At 24 h, the elevation in β-endorphin levels in the mPFC was completely absent in the ketamine group (Fig. 3E), and there was no change in *Pomc* mRNA expression in the hypothalamus (Fig. 3F); however, hypothalamic *Pomc* expression remained positively correlated with β-endorphin level in the mPFC (Fig. 3G). Ketamine treatment did not significantly change the mRNA expression of *Oprm1* (Fig. 3H), *Pomc* (Supplementary Fig. S3E), or *Oprk1* (Supplementary Fig. S3F) in the mPFC at 24 h. Thus, ketamine induced a rapid, non-sustained increase in β-endorphin and the expression of the gene that encodes its primary receptor, µ-OR, in the mPFC, and the expression of the gene that encodes the β-endorphin precursor, POMC, in the hypothalamus.

### Intra-mPFC anti-β-endorphin antibody infusion blocks the antidepressant-like actions of ketamine

**Fig. 4:**
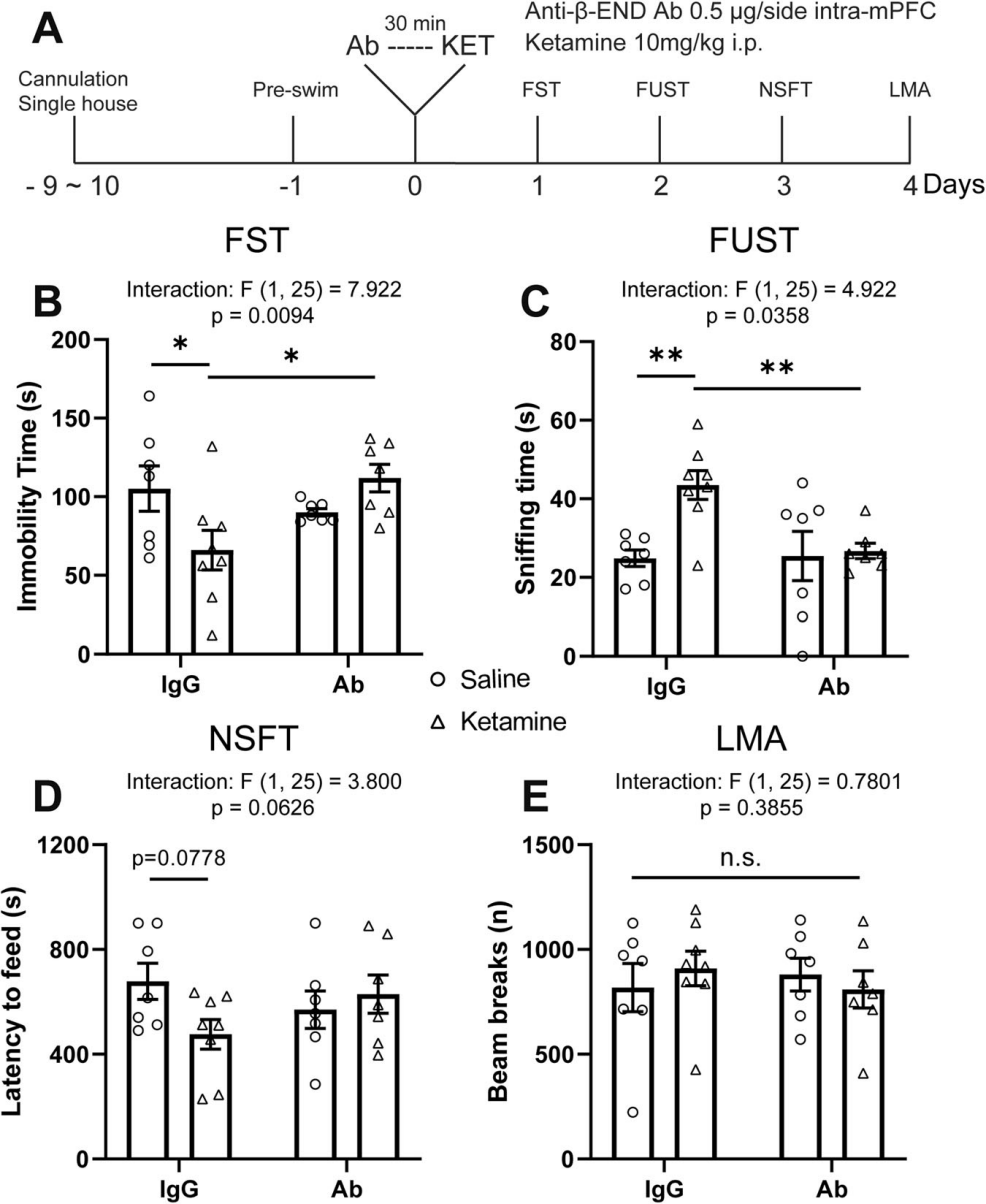
β-endorphin neutralization in the mPFC blocks the antidepressant-like actions of ketamine. **A** Schematic timeline of intra-mPFC anti-β-endorphin neutralizing antibody pretreatment, ketamine treatment, and behavioral testing. Intra-mPFC anti-β-endorphin neutralizing antibody pretreatment blocks (**B**) the reduction in immobility time in the FST (ketamine x antibody interaction: *F*_(1, 25)_ = 7.922, *p* = 0.0094), **C** the increase in sniffing time in the FUST (interaction: *F*_(1,25)_ = 4.922, *p* = 0.0358), **D** the trend-level reduction of latency to feed in the NSFT induced by ketamine (interaction: *F*_(1, 25)_ = 3.800, *p* = 0.0626), without affecting locomotor activity (interaction: *F*_(1, 25)_ = 0.7801, *p* = 0.3855) (**E**). Two-way ANOVA followed by Sidak’s post hoc test. *n* = 7 for IgG/SAL, *n* = 8 for IgG/KET, *n* = 7 for Ab/SAL, *n* = 7 for Ab/KET. Post hoc significant effects indicated as: *p < 0.05, **p < 0.01. ns nonsignificant, IgG control IgG, Ab anti-β-endorphin neutralizing antibody, FST forced swim test, FUST female urine sniffing test, NSFT novelty suppressed feeding test, LMA locomotion.

We next tested whether β-endorphin in the mPFC is necessary for the behavioral effects of ketamine. We infused rats with a β-endorphin neutralizing antibody (0.5 μg/0.5 μl/side) 30 minutes before ketamine treatment (10 mg/kg, i.p.) and subjected them to behavioral tests starting 24 hours later (Fig. 4A). In control IgG-infused rats, ketamine produced robust behavioral effects that were lost in the rats that received β-endorphin antibody infusion: FST (Fig. 4B), FUST (Fig. 4C) and NSFT (Fig. 4D). There was no effect on locomotor activity or home cage feeding (Fig. 4E and Supplementary Fig. S4). Intra-mPFC β-endorphin antibody infusion alone did not influence these behaviors.

### Systemic naltrexone pretreatment abolishes ketamine-induced molecular changes in the mPFC

**Fig. 5:**
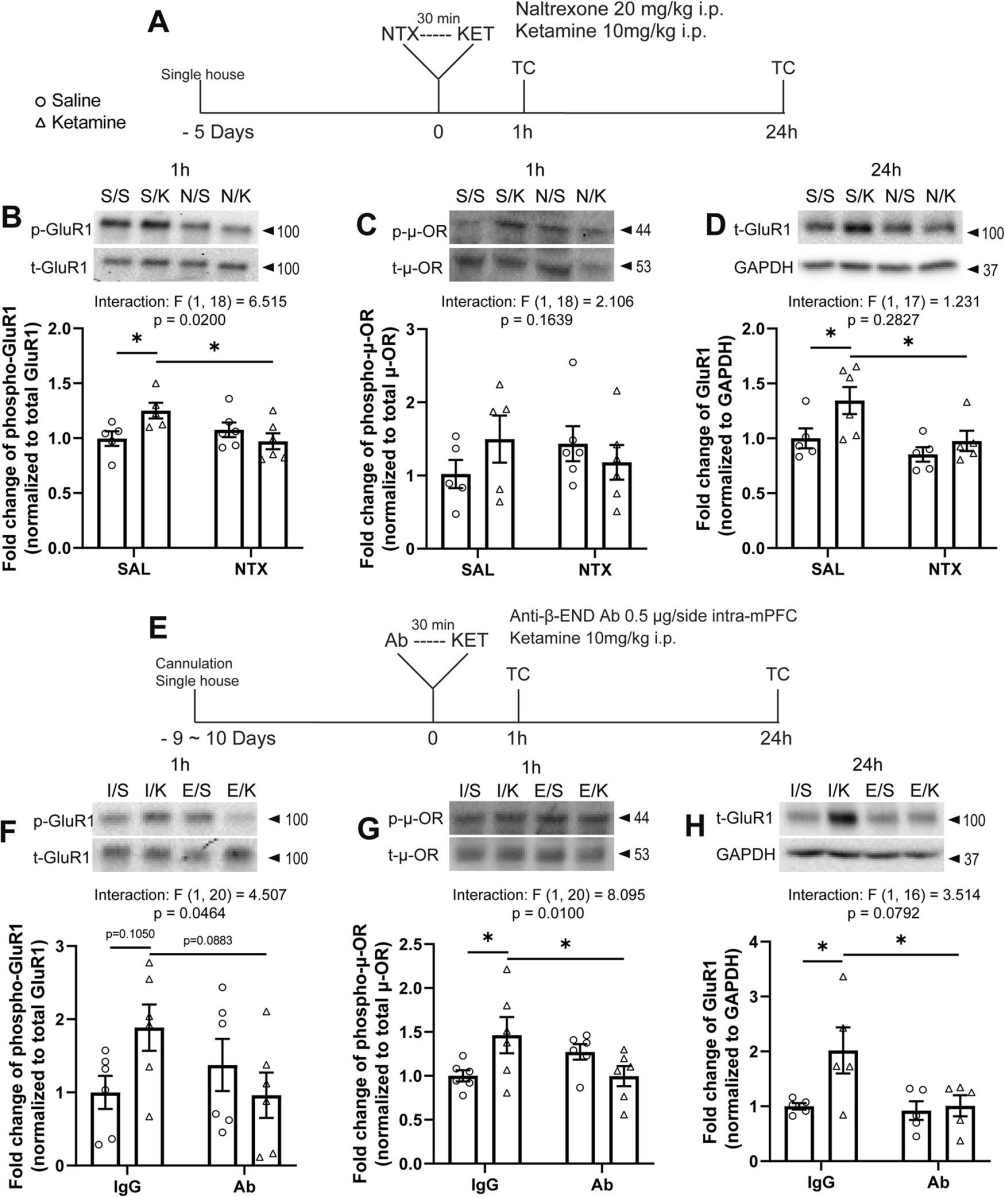
Systemic naltrexone pretreatment and intra-mPFC anti-β-endorphin antibody infusion block the molecular effects of ketamine. **A** Schematic timeline of systemic naltrexone pretreatment, ketamine treatment, and tissue collection. Systemic naltrexone pretreatment blocks (**B**) the increase in phosphorylation of GluR1 (interaction: *F*_(1, 18)_ = 6.515, *p* = 0.0200) and (**C**) the trend-level increase in phosphorylation of μ-ORs at Ser375 in the total homogenate induced by ketamine at 1 h. **D** Systemic naltrexone pretreatment blocks total GluR1 levels in the synaptosomes induced by ketamine at 24 h (ketamine main effect: *F*_(1, 17)_ = 5.492, *p* = 0.0315; naltrexone main effect: *F*_(1, 17)_ = 6.673, *p* = 0.0193). **E** Schematic timeline of intra-mPFC anti-β-endorphin neutralizing antibody pretreatment, ketamine treatment, and tissue collection. Intra-mPFC anti-β-endorphin neutralizing antibody pretreatment blocks (**F**) the trend-level increase in GluR1 phosphorylation (interaction: *F*_(1, 20)_ = 4.507, *p* = 0.0464) and (**G**) the increase in phosphorylation of μ-ORs at Ser375 (interaction: *F*_(1, 20)_ = 8.095, *p* = 0.0100) in the total homogenate at 1 h. **H** Intra-mPFC anti-β-endorphin neutralizing antibody pretreatment blocks the increase in total GluR1 levels in the synaptosomes at 24 h (interaction: *F*_(1, 16)_ = 3.514, *p* = 0.0792; ketamine main effect: *F*_(1, 16)_ = 4.980, *p* = 0.0403; antibody main effect: *F*_(1, 16)_ = 4.809, *p* = 0.0434). Two-way ANOVA followed by Sidak’s post hoc test. *n* = 5 for S/S and S/K, *n* = 6 for N/S and N/K in **B** and **C**; *n* = 5 for S/S, N/S, N/K and *n* = 6 for S/K in **D**; *n* = 6/group in **F** and **G**; *n* = 5/group in **H**. Post hoc significant effects indicated as: *p < 0.05. SAL/S saline, NTX/N naltrexone, K ketamine, IgG/I control IgG, Ab/E anti-β-endorphin neutralizing antibody.

To determine the molecular mechanisms underlying impaired ketamine response following systemic naltrexone pretreatment, we examined the effect of naltrexone pretreatment on ketamine-induced phosphorylation of AMPA receptor subunit GluR1, which has been implicated in ketamine’s antidepressant-like actions [2, 36, 37], using Western blotting. mPFC tissue was collected 1 h after ketamine injection, preceded by naltrexone or saline (Fig. 5A). Ketamine significantly increased phosphorylation of GluR1 in the total homogenate (Fig. 5B); this was blocked by naltrexone pretreatment. Similarly, ketamine induced a numerically increased phosphorylation of μ-OR, which was partially blocked by naltrexone pretreatment at 1 h, though these effects did not reach statistical significance (Fig. 5C).

We next determined the effect of naltrexone pretreatment on the ketamine-induced elevation in new synthesis of GluR1 in the mPFC [12, 38]. 24 h after ketamine treatment, GluR1 protein levels were significantly increased in mPFC synaptosomes in saline-pretreated rats, while no significant changes were observed in naltrexone-pretreated group (Fig. 5D). These results indicate that opioid receptor activation is required for synaptic changes induced by ketamine in the mPFC.

### Intra-mPFC anti-β-endorphin antibody infusion blocks the molecular effects of ketamine

To determine whether β-endorphin in the mPFC is required for these molecular effects, we infused rats with a β-endorphin neutralizing antibody 30 min before ketamine treatment and collected mPFC 1 h and 24 h after ketamine (Fig. 5E). Increased phosphorylation of GluR1 (Fig. 5F) and μ-OR (Fig. 5G) in the total homogenate at 1 h were both absent in rats pretreated with β-endorphin antibody. Increased GluR1 levels in synaptosomes at 24 h (Fig. 5H) was similarly abolished by β-endorphin antibody pretreatment.

## Discussion

We demonstrate that blockade of opioid receptors by a single dose of systemic naltrexone 30 min prior to ketamine treatment abolishes the effects of ketamine on behavioral despair, anhedonia, and anxiety-like phenotypes in rats. This is consistent with several previous clinical and preclinical reports [14, 15, 17–19], though not with others [16, 20]. These discrepancies in the literature could be explained by the differences in the routes and doses of naltrexone administered, the timepoints at which naltrexone is administered, comorbidity of alcohol use disorder with depression in one clinical study [16], and prior exposure to stress, which influences expression of opioid receptor [39], in one preclinical study [20]. As stressed animals may provide better predictive validity in studies of behavioral models of depression-like behaviors, future preclinical studies employing stress exposure, comprehensive behavioral phenotyping, and various dosing of naltrexone are needed to clarify these discrepancies.

Intra-mPFC infusion of naltrexone prior to ketamine treatment also blocks the behavioral effects of ketamine, and systemic naltrexone pretreatment blocks ketamine-induced molecular changes in the mPFC. These results indicate that opioid signaling in the mPFC plays a central role in regulating ketamine’s actions. This importantly extends recent research on the interplay between ketamine and the opioid system by pinpointing a critical site of action, although our current data do not exclude contributions from other locations.

The naltrexone dose (20 mg/kg) used in our study is relatively high compared to those used in other preclinical studies (1 mg/kg [17, 19], 2 mg/kg [18], 10 mg/kg [20]). The species and strain of animals (congenital learned helplessness (cLH) rats for [17] and C57BL/6 mice for [18, 19] and the drug treatment protocol (naltrexone and ketamine injections were given three times before FST in ref. [19]) also differ between these past studies and the current work. It is possible that lower doses of naltrexone would be sufficient to block ketamine’s effects in our paradigm. Naltrexone can bind to μ-ORs, κ-ORs, and δ-ORs at the dose we used [24–27], and β-endorphin binds to both μ-ORs and δ-ORs, with a lower affinity at κ-ORs. Recent studies have reported that pharmacological blockade of κ-ORs abolished the behavioral effects of repeated ketamine administration in the FST in mice [19], and that δ-OR agonists produce antidepressant-like effects [40]. Although ketamine-induced phosphorylation of μ-ORs and upregulation of *Pomc* suggest a μ-OR-mediated mechanism, we cannot rule out the contribution of κ-ORs and δ-ORs in mediating ketamine’s actions. Future studies using lower doses of naltrexone and/or more specific antagonists for μ-ORs, κ-ORs, and δ-ORs and more comprehensive behavioral testing and molecular characterization will be needed to unambiguously determine the specific type(s) of ORs that mediate ketamine’s actions.

Our results support an intriguing model that merits further study: ketamine induces glutamate release by blocking NMDARs on GABAergic interneurons [41], resulting in disinhibition and a burst of glutamate [42, 43], leading to increased release of β-endorphin from the axon terminals of ARC POMC neurons in the mPFC [44]; this is similar to ketamine-induced release of dopamine, serotonin and noradrenaline [42, 45, 46], which all show rapid elevation within an hour following ketamine administration. Of note, we did not directly test the effects of NMDAR inhibition on β-endorphin release in the mPFC. Replicating these experiments using other NMDAR antagonists could clarify whether NMDAR inhibition, as opposed to some other effects of ketamine, causes the release of β-endorphin in the mPFC. Regardless, released β-endorphin then activates μ-OR on GABAergic interneurons and/or astrocytes [47, 48]. Inhibition of interneurons and release of glutamate from astrocytes, both of which are known effects of μ-OR activation [49–52] could synergize with the direct effects of ketamine on GABAergic interneurons and reinforce the surge in synaptic glutamate [41–43], thus contributing to the initiation of rapid and sustained antidepressant-like effects. Alternatively, ketamine could stimulate β-endorphin release in the hypothalamus, which may then be transported via cerebrospinal fluid in the ventricular system, arriving at the mPFC within an hour [53]. These two mechanisms could also work synergistically, with ketamine-induced local β-endorphin release in the mPFC exerting immediate actions while β-endorphin transported via volume transmission influences the mPFC for a longer period of time, perhaps sustaining the earlier neuronal effects.

Ketamine may have direct actions other than the blockade of NMDA receptors. For example, the ketamine metabolite (2R,6R)-hydroxynorketamine (HNK) produces antidepressant-relevant effects in the absence of direct inhibition of NMDAR [54, 55]. One recent study reported that HNK acts as an inverse agonist of both μ-OR and κ-OR [56]. Another important molecular pathway implicated in the ketamine’s actions is BDNF/TrkB signaling. BDNF expression and BDNF release in the mPFC are required for ketamine’s antidepressant actions [57, 58]. Interestingly, a recent study suggested that ketamine can directly bind to TrkB, thus promoting BDNF signaling [59]. As POMC neurons in the ARC express TrkB [60], direct binding of TrkB by ketamine could lead to activation of POMC neurons, which may explain the increased *Pomc* mRNA expression that we observe. Activation of BDNF/TrkB signaling may de-suppress translation via mTOR and/or eEF2 pathways, leading to rapid synthesis of proteins and peptides [61], including β-endorphin, which could be transported to and released by axon terminals in the mPFC. Future studies testing the relationship of OR-mediated effects on these initial molecular pathways are warranted.

Although ketamine has an appreciable binding affinity for both μ-ORs and κ-ORs [3, 4], our β-endorphin antibody neutralization data suggest that mPFC OR activation following ketamine depends on β-endorphin in the mPFC, not direct effects of ketamine on ORs.

We find evidence for stimulation of β-endorphin release by ketamine in hypothalamic neuronal culture (Supplementary Fig. S3A, B), as has previously been reported in pituitary cell culture [62]. Previous studies suggest that ketamine induces endogenous opioid release in vivo [63, 64]. We document elevated β-endorphin levels in the mPFC, and a correlated increase in *Pomc* mRNA levels in the hypothalamus, 1 h following ketamine treatment in vivo; both parameters are unchanged at 24 h but remain correlated with one another. *Pomc* mRNA levels in the mPFC are not significantly changed by ketamine treatment at both 1-hour and 24-hour timepoints. These observations suggest that ketamine activates ARC POMC neurons, directly or indirectly and perhaps in conjunction with other processes [41–43], leads to rapid and transient increase in β-endorphin in the mPFC, initiating the antidepressant-like effects.

β-endorphin has been implicated in the pathophysiology and treatment of depression [8, 9]. Plasma β-endorphin levels are correlated with certain clinical symptoms of depression [65], and several antidepressant treatments increase β-endorphin levels in plasma [66, 67]. Release of endogenous opioid(s) targeting μ-ORs in the PFC has been observed after high-intensity exercise [68], which has been linked to improved mood [69]. Fluoxetine, a selective serotonin reupdate inhibitor, has been demonstrated to induce β-endorphin release in the ARC and nucleus accumbens [70]. Although our observations that ketamine increases β-endorphin levels in the mPFC in vivo and that intra-mPFC pretreatment with anti-β-endorphin neutralizing antibody blocks the antidepressant-like effects of ketamine suggest a causal relationship between β-endorphin in the mPFC and ketamine’s actions, we did not directly monitor changes in the extracellular level of β-endorphin in the mPFC in response to ketamine (the increase shown in Fig. 3A was in total mPFC tissue), nor did we examine potential ketamine-induced β-endorphin release in other brain regions. Future study using in vivo microdialysis could better characterize the temporal profile of ketamine-induced β-endorphin release in the mPFC. Future examination of multiple brain regions might also identify other regions where β-endorphin may have a role in mediating ketamine’s actions.

Previous studies have implicated brain-derived neurotrophic factor (BDNF) and vascular endothelial growth factor (VEGF) in the antidepressant-like actions of ketamine [58, 71]. Whether these trophic factors and their respective signaling act in parallel with or subsequently to β-endorphin remains to be determined. β-endorphin has been reported to increase BDNF expression in the PFC and hippocampus [72], and μ-OR agonists activate VEGF receptors [73], suggesting the possibility that BDNF and VEGF signaling could be downstream of β-endorphin. However, it is equally possible that ketamine induces release of β-endorphin, BDNF, and VEGF independently and they then act interdependently, together with other processes, to mediate ketamine’s antidepressant-like effects.

It has been shown that ketamine and other agents with rapid antidepressant-like properties rapidly induces GluR1 phosphorylation in multiple brain regions, including mPFC [2, 36–38, 61], and that GluR1 phosphorylation is required for the rapid and sustained antidepressant-like effects of ketamine and subsequent increase in synaptic GluR1 levels [36]. Our results indicate that activation of opioid receptors and presence of β-endorphin in the mPFC are required for ketamine-induced increase in GluR1 phosphorylation and elevated synaptosomal GluR1 levels in the mPFC. μ-OR agonists have been shown to increase protein kinase A and calcium/calmodulin-dependent protein kinase II activity in vivo [74, 75], which can in turn phosphorylates GluR1 [76, 77], mediating its role in regulating synaptic delivery, and incorporation of GluR1-containing AMPA receptors into synapses [78]. β-endorphin leads to phosphorylation of μ-ORs at Ser375 [79]. We show that ketamine increases phosphorylation of μ-ORs at Ser375 in the mPFC in a β-endorphin-sensitive manner. This observation suggests that μ-OR activation in the mPFC is due to ketamine-induced elevation of β-endorphin and is not consequence of a direct action of ketamine.

Preclinical studies have begun to reveal sex differences in response to ketamine. Females are sensitive to lower dose of ketamine and exhibit stronger behavioral response in some contexts [80]. Sex differences have also been reported in β-endorphin levels in multiple brain regions, both at baseline and under various experimental conditions [81, 82]. Because of these reported effects of sex, we focused here on male rats, to reduce the number of variables at play. It will be important to examine potential sexual dimorphisms in the reported effects in future studies.

Our data suggest that β-endorphin in the mPFC can contribute to antidepressant-like effects. Previous studies have provided conflicting evidence on this question. In mice, morphine reduces immobility time in the FST and tail suspension test (TST) [83, 84]. In rats, however, morphine does not influence the immobility time in the FST [17, 85]. Early clinical studies documented antidepressant effects induced by intravenous β-endorphin infusion [86–88]. Within central nervous system, intracerebroventricular infusion of β-endorphin increases *Bdnf* mRNA expression in the PFC and hippocampus [72], similar to the effects seen following chronic conventional antidepressant treatments [89] and acute ketamine administration [90]. Interestingly, one recent study found that endogenous and exogenous opioids act on GABAergic and glutamatergic neurons, respectively, to mediate behavioral effects [91]. Therefore, the lack of consistent effects from exogenous μ-OR agonists cannot rule out the possibility that endogenous β-endorphin possesses rapid antidepressant potential.

In summary, our study demonstrates that β-endorphin and opioid receptor activation in the mPFC are required for the behavioral and molecular actions of ketamine in a well-established rat model. These findings are consistent with accumulating evidence implicating endogenous opioid signaling in the rapid antidepressant effects of ketamine. Importantly, our results suggest a potential mechanism by which ketamine produces antidepressant-like actions: by increasing β-endorphin release, which in turn activates μ-ORs in the mPFC. This work lays the foundation for future studies to further delineate these mechanisms to inform the development of next-generation rapid-acting antidepressant agents.

### Supplementary information

Supplemental material

Supplemental Table 1

## Data Availability

Raw data from these experiments will be made available for secondary analysis by academic investiagors upon request.
