# Supplementary material for: The endogenous opioid system in the medial prefrontal cortex mediates ketamine’s antidepressant-like actions
Source: Transl Psychiatry. 2024 Feb 12;14:90. doi: 10.1038/s41398-024-02796-0 (PMC10861497; doi:10.1038/s41398-024-02796-0)
Supplement: Supplementary file 1 — Supplemental material [file 41398_2024_2796_MOESM1_ESM.docx]

**SUPPLEMENTAL MATERIAL**

**Supplementary Materials and Methods**

### Western blot

### Tissue preparations and Western blots were conducted as previously described [1]. mPFC from adult rats was dissected from coronal sections and samples were homogenized in a solution containing 0.32 M sucrose, 20 mM HEPES (pH 7.4), 1 mM EDTA, 1× protease inhibitor cocktail, 5 mM NaF, and 1 mM sodium vanadate. The homogenate was centrifuged for 10 minutes at 900 g at 4°C, and the supernatant was centrifuged at 16,000 g for 10 minutes. After centrifugation, the supernatant (cytosolic fraction – S1) was removed and the pellet (crude synaptosomal fraction – P2) was resuspended by sonication in protein lysis buffer (50 mM Tris-HCl [pH 7.5], 150 mM NaCl, 1% Triton X-100, 0.1% SDS, 2 mM EDTA, 1 mM NaVO3, 5 mM NaF, and 1× protease inhibitor cocktail). To prepare total homogenate, samples were homogenized in aforementioned protein lysis buffer. The homogenate was centrifuged for 10 minutes at 16,000 g, and supernatant was collected. Protein concentration was determined by Pierce BCA Protein Assay Kit (Thermo Scientific). For Western blotting, equal amounts of protein (5 ~ 10 μg) for each sample were loaded into 4%–20% or 7.5% SDS PAGE gel for electrophoresis. PVDF membranes with transferred proteins were blocked with 5% BSA or 5% skim milk in TBST (TBS + 0.1% Tween-20) for 1 hour at room temperature and incubated with primary antibodies overnight at 4°C. The following primary antibodies were used: phospho-OPRM1 (Ser375, Bioss, #BS3742R, 1:1000), total OPRM1 (Thermo Scientific, #PA124628, 1:1000), phospho-GluR1 (Ser845, Cell Signaling, #8084, 1:1000), total GluR1 (Cell Signaling, #13185, 1:1000 or DSHB, #N355/1, 1:100), and GAPDH (Cell Signaling, #2118, 1:2000 or Millipore-Sigma, #MAB374, 1:2000). The next day, blots were washed 3 times in TBST and incubated with horseradish peroxidase–conjugated anti-rabbit or anti-mouse secondary antibody (Vector Laboratories, #PI-1000-1 or #PI-2000-1, 1:5000 to 1:10000) for 1 hour at room temperature. After 3 washes with TBST, bands were detected using ECL (Thermo Scientific). The blots then were incubated in stripping buffer (Thermo Scientific, #46430) for 15 minutes at room temperature, followed by extensive washes with TBST, and reused to detect other proteins. Densitometric analysis of immunoreactivity for each protein was conducted using Image Lab (Bio-Rad, Hercules, California). Immunoreactivity was normalized to saline-treated control group values for each protein.

**RNA extraction and qPCR analysis**

# RNA from rat tissue specimens, obtained by dissection (mPFC and hypothalamus), was extracted using the RNeasy Mini Kit (Qiagen, Hilden, Germany) according to the manufacturer’s protocol, and 0.5 µg was reverse transcribed using iScript cDNA Synthesis Kit (Bio-Rad) or High Capacity cDNA Reverse Transcription Kit (Thermo Scientific). 10 ng of first-strand cDNA was subjected to PCR amplification using iTaq Universal SYBR Green Supermix (Bio-Rad) or PowerUp SYBR Green Master Mix (Applied Biosystems). Each reaction was performed in duplicate and ΔΔCt method was used to quantify relative gene expression normalized to glyceraldehyde 3-phosphate dehydrogenase (GAPDH). Primers for rat *Pomc*-forward, 5’- GGTCCCTCCAATCTTGTTTGC-3’, rat *Pomc*-reverse, 5’- ACTGTAGCAGAATCTCGGCATCT-3’, rat *Oprm1*-forward, 5’-CCGAAATGCCAAAATCGTCA-3’, rat *Oprm1*-reverse, 5’-GGACCCCTGCCTGTATTTTGT-3’, rat *Oprk1*-forward, 5’- TCTAGCTATTACTTCTGCATTG-3’, rat *Oprk1*-reverse, 5’- TGTGTTTCTAACTCTGTTTGT-3’, rat *Gapdh*-forward, 5’-GAACATCATCCCTGCATCCA -3’, rat *Gapdh*-reverse, 5’-CCAGTGAGCTTCCCGTTCA -3’.

### Primary hypothalamic culture and *in vitro* ketamine treatment

Pregnant females were euthanized with isoflurane and hypothalamus were dissected from E18 embryos. After incubation in trypsin-EDTA (0.25%; Gibco) for 10 min, hypothalami were dissociated and neurons were plated at 0.6 million cells per well in 6-well polylysine-coated plates in DMEM (Gibco) containing 10% fetal bovine serum and 1% penicillin-streptomycin. The following day, the medium was changed to a serum-free medium containing neurobasal and B27 (Gibco), which was changed every 3 days. Cells were maintained at 37 °C, 5% CO_2_, and 95% humidity. After 12 days *in vitro* the medium was changed to fresh neurobasal medium. Four hours following the medium change, cultured neurons were incubated with 0.5 µM ketamine for 60 min. Following the incubation with ketamine, the media were collected and stored at – 80 °C until ELISA analysis.

**Supplementary Figures**


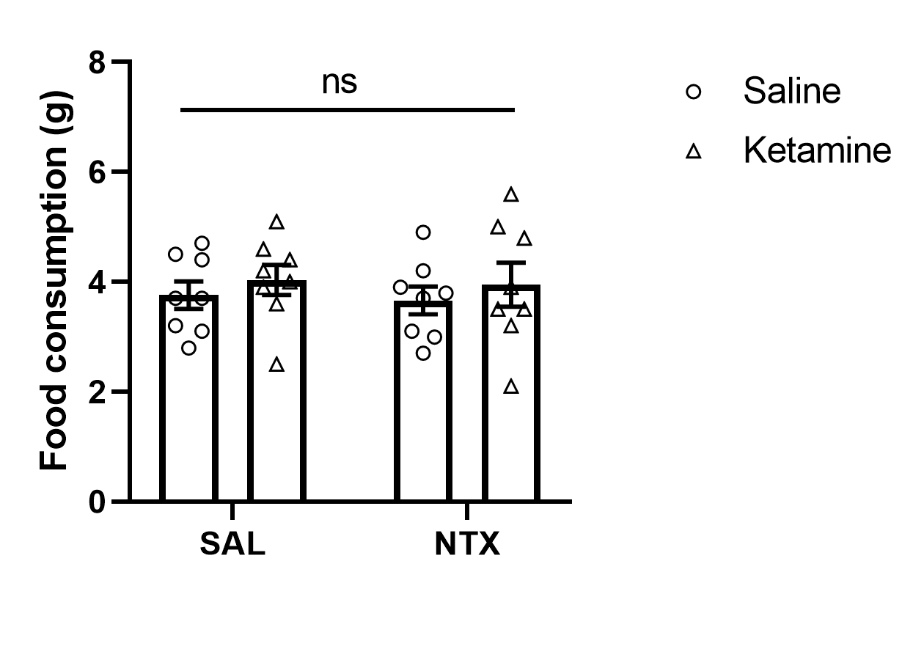


**Figure S1A**, related to Figure 1D**.** Systemic naltrexone pretreatment and subsequent ketamine treatment do not influence home cage food consumption (ketamine x naltrexone interaction: F_(1, 28)_ = 0.00043, p = 0.9835). Two-way ANOVA followed by Sidak’s post hoc test. n = 8/group. ns, nonsignificant; SAL, saline; NTX, naltrexone.


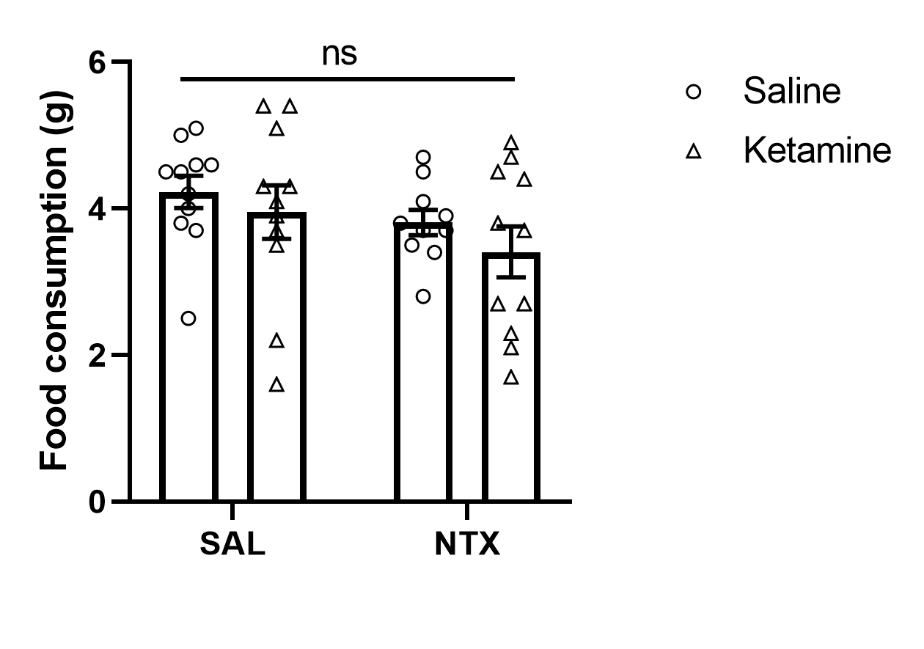


**Figure S2A**, related to figure 2D**.** Intra-mPFC naltrexone pretreatment and subsequent ketamine treatment do not influence home cage food consumption (ketamine x naltrexone interaction: F_(1, 39)_ = 0.04807, p = 0.8276). Two-way ANOVA followed by Sidak’s post hoc test. n = 11/group for SAL/SAL, SAL/KET, and NTX/KET; n = 10 for NTX/SAL. ns, nonsignificant; SAL, saline; NTX, naltrexone.


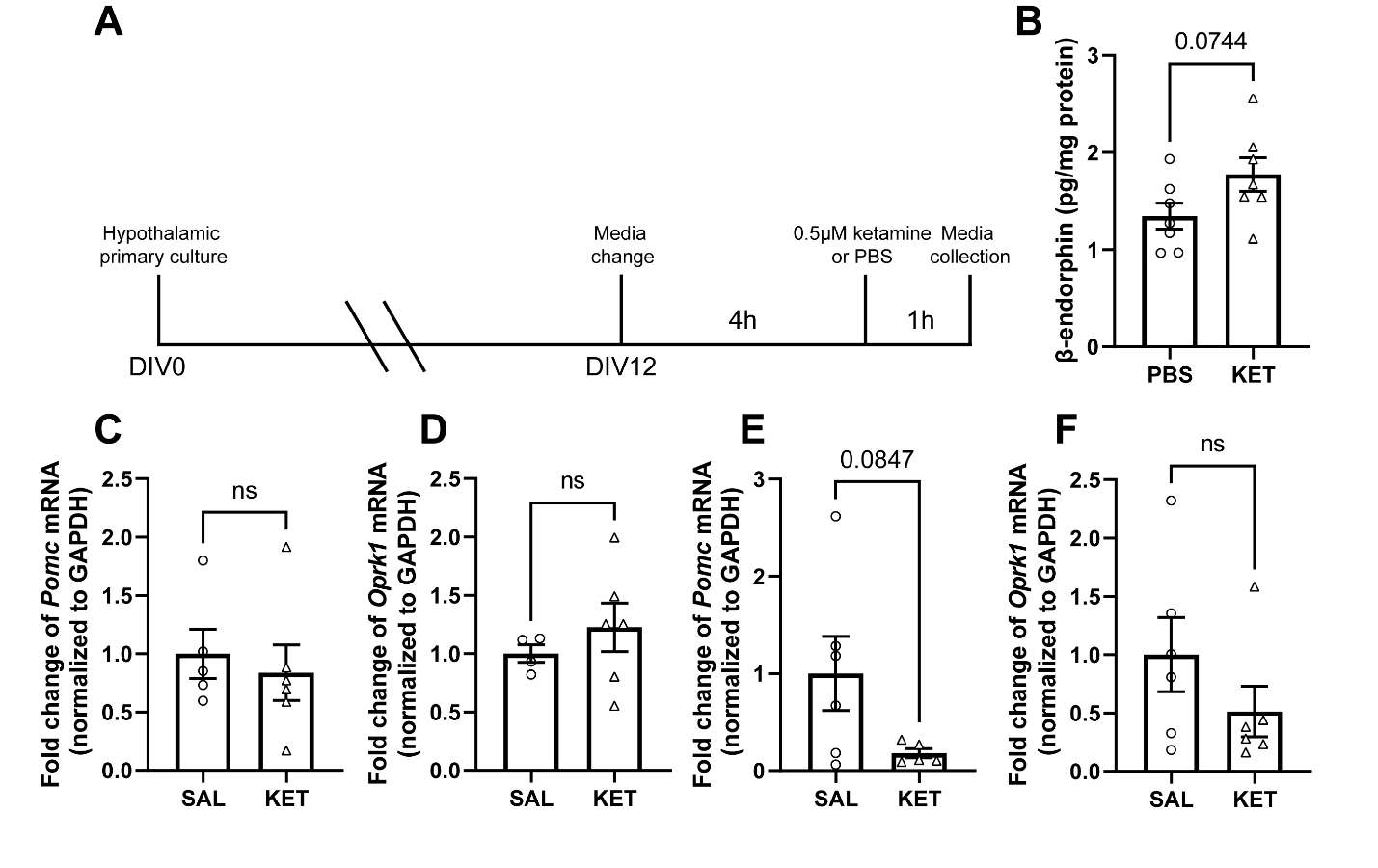


**Figure S3**, related to Figure 3. (**A**) Schematic timeline of hypothalamic primary culture, ketamine treatment, and media collection. (**B**) *In vitro* Ketamine (0.5μM) treatment increases β-endorphin release in the hypothalamic primary culture at 1h, at trend level (t_(12)_ = 1.954, p = 0.0744). *In vivo* ketamine (10 mg/kg, i.p.) treatment does not significantly change the *Pomc* (t_(9)_ = 0.4997, p = 0.6293) and *Oprk1* (t_(8)_ = 0.8456, p = 0.4224) mRNA expression in the mPFC at (**C, D**)1h and at (**E, F**) 24h (*Pomc*: (t_(5.158)_ = 2.130, p = 0.0847; *Oprk1*: t_(10)_ = 1.263, p = 0.2353). Student’s t test. n = 7/group in B; n = 5 for SAL, n = 6 for KET in C; n = 4 for SAL, n = 6 for KET in D; n = 6 for SAL, n = 5 for KET in E; n = 6/group in F.


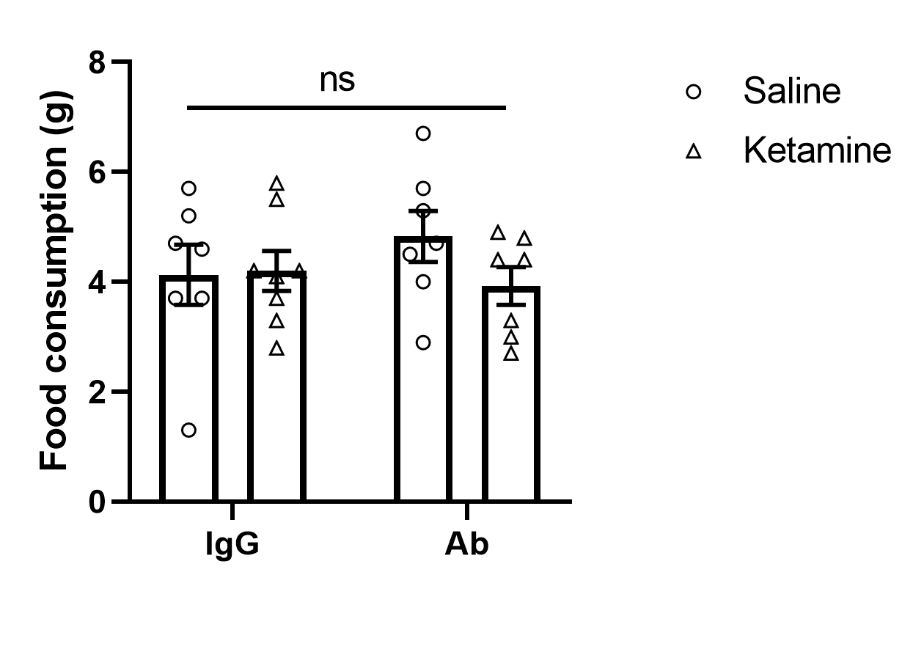


**Figure S4A**, related to Figure 4D**.** Intra-mPFC anti-β-endorphin neutralizing antibody pretreatment and subsequent ketamine treatment do not influence home cage food consumption (ketamine x antibody interaction: F_(1, 25)_ = 1.263, p = 0.2718). Two-way ANOVA followed by Sidak’s post hoc test. n = 7 for IgG/SAL, n = 8 for IgG/KET, n = 7 for Ab/SAL, n = 7 for Ab/KET. ns, nonsignificant; IgG, control IgG; Ab, anti-β-endorphin neutralizing antibody.

**REFERENCES**

1. Li N, Lee B, Liu RJ, Banasr M, Dwyer JM, Iwata M, et al. mTOR-dependent synapse formation underlies the rapid antidepressant effects of NMDA antagonists. Science. 2010;329(5994):959-64. doi:10.1126/science.1190287
